# Visualizing hierarchies in scRNA-seq data using a density tree-biased autoencoder

**DOI:** 10.1093/bioinformatics/btac249

**Published:** 2022-06-27

**Authors:** Quentin Garrido, Sebastian Damrich, Alexander Jäger, Dario Cerletti, Manfred Claassen, Laurent Najman, Fred A Hamprecht

**Affiliations:** HCI/IWR, Heidelberg University, 69120 Heidelberg, Germany; Université Gustave Eiffel, CNRS, LIGM, F-77454 Marne-la-Vallée, France; HCI/IWR, Heidelberg University, 69120 Heidelberg, Germany; HCI/IWR, Heidelberg University, 69120 Heidelberg, Germany; Institute of Molecular Systems Biology, ETH Zürich, 8093 Zürich, Switzerland; Institute of Microbiology, ETH Zürich, 8093 Zürich, Switzerland; Internal Medicine I, University Hospital Tübingen, Faculty of Medicine, University of Tübingen, 72076 Tübingen, Germany; Université Gustave Eiffel, CNRS, LIGM, F-77454 Marne-la-Vallée, France; HCI/IWR, Heidelberg University, 69120 Heidelberg, Germany

## Abstract

**Motivation:**

Single-cell RNA sequencing (scRNA-seq) allows studying the development of cells in unprecedented detail. Given that many cellular differentiation processes are hierarchical, their scRNA-seq data are expected to be approximately tree-shaped in gene expression space. Inference and representation of this tree structure in two dimensions is highly desirable for biological interpretation and exploratory analysis.

**Results:**

Our two contributions are an approach for identifying a meaningful tree structure from high-dimensional scRNA-seq data, and a visualization method respecting the tree structure. We extract the tree structure by means of a density-based maximum spanning tree on a vector quantization of the data and show that it captures biological information well. We then introduce density-tree biased autoencoder (DTAE), a tree-biased autoencoder that emphasizes the tree structure of the data in low dimensional space. We compare to other dimension reduction methods and demonstrate the success of our method both qualitatively and quantitatively on real and toy data.

**Availability and implementation:**

Our implementation relying on PyTorch and Higra is available at github.com/hci-unihd/DTAE.

**Supplementary information:**

Supplementary data are available at *Bioinformatics* online.

## 1 Introduction

Single-cell RNA sequencing (scRNA-seq) data allows analyzing gene expression profiles at the single-cell level, thus granting insights into cell behavior at unparalleled resolution. In particular, this permits studying the cell development through time more precisely.

Waddington’s popular metaphor likens the development of cells to marbles rolling down a landscape (Waddington, 1957). While cells are all grouped at the top of the hill when they are not yet differentiated (e.g., stem cells), as they start rolling down, they can take multiple paths and end up in distinct differentiated states, or cell fates.

However, for every cell, hundreds or thousands of expressed genes are recorded, and this data are noisy. To summarize such high-dimensional data, it is useful to visualize it in two or three dimensions.

**Fig. 1.**
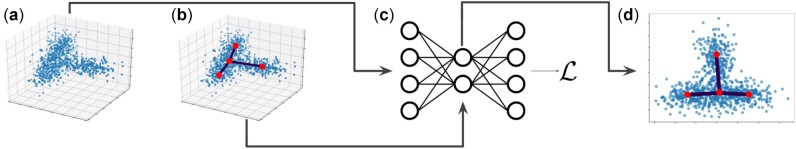
Schematic method overview. (**a**) High-dimensional data. (**b**) Proposed density tree. After computing the *k*-means centroids on the data, we build a tree based on the data density between pairs of centroids. (**c**) DTAE. An autoencoder is used to learn a representation of our data. This embedding is regularized by the previously computed tree in order to preserve its hierarchical structure in low-dimensional space. (**d**) The final DTAE embedding. After training of the autoencoder, the bottleneck layer visualizes the data in low dimension and respects the density structure

Our goal, then, is to identify the hierarchical (tree) structure of the scRNA-seq data and subsequently reduce its dimensionality while preserving the extracted hierarchical properties. We address this in two steps, illustrated in Figure 1.

First, we cluster the scRNA-seq data in high-dimensional space to obtain a more concise and robust representation. Then, we capture the hierarchical structure as a minimum spanning tree (MST) on our cluster centers, with edge weights reflecting the data density in high-dimensional space. We dub the resulting tree ‘density tree’.

Second, we embed the data to low dimension with an autoencoder (AE), a type of artificial neural network. In addition to the usual aim of reconstructing its input, we bias the AE to also reproduce the density tree in low-dimensional space. As a result, the hierarchical properties of the data are emphasized in our visualization.

## 2 Related work

There are various methods for visualizing scRNA-seq data and trajectory inference, and many of them have been reviewed for instance in Saelens *et al.* (2019). We therefore mention only some exemplary approaches here.

**Fig. 2.**
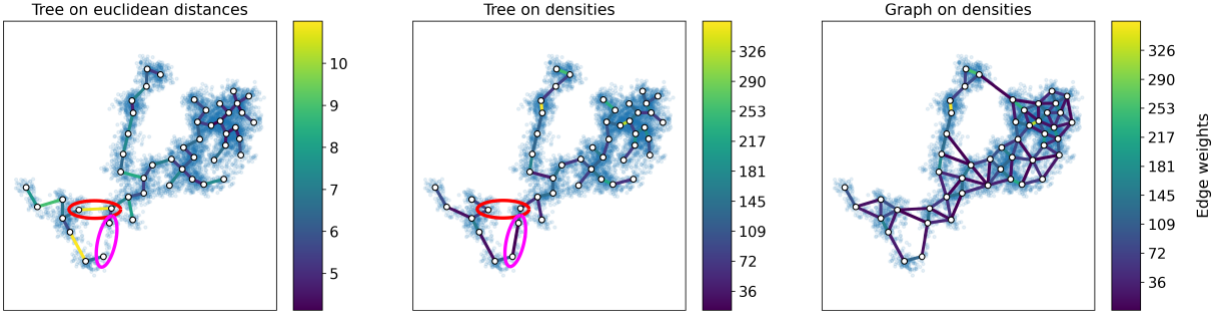
(left, middle) Comparison of the tree built on *k*-means centroids using Euclidean distance or density weights. The data were generated using the PHATE library (Moon *et al.*, 2019), with three branches in 2D. Original data points are transparently overlayed to better visualize their density. While the tree based on the Euclidean distance places connections between centroids that are close but have only few data points between them (see topmost ellipse), our tree based on the data density instead includes those edges that lie in high-density regions (see bottommost ellipse). (right) Complete graph over centroids and its Hebbian edge weights. Null-weight edges, that is edges not supported by data, are omitted for clarity

**Graph only:** SCORPIUS (Cannoodt *et al.*, 2016) was one of the first such methods. It is limited to linear topologies rather than trees. More versatile methods include SLINGSHOT (Street *et al.*, 2018) and SPADE (Bendall *et al.*, 2011; Qiu *et al.*, 2011). In contrast to our work, these three methods only provide a graph summary of the data, but not a 2D scatter plot. Similar to our density tree, SLINGSHOT and SPADE determine the hierarchical structure of the dataset as an MST on cluster centers. However, SLINGSHOT does not consider density. SPADE addresses the data density only by downsampling dense regions to equalize the data density. In particular, it does not inform the MST by the actual data density, which can be problematic, as illustrated in Figure 2. In contrast, we induce our density tree to have edges in high-density regions. PAGA (Wolf *et al.*, 2019) produces primarily a graph summary of the data. It first clusters the *k* nearest neighbor (*k*NN) graph of the data by modularity, and then places edges between clusters of high connectivity. Optionally, a layout of the PAGA graph can serve as initialization to other methods, such as UMAP. In our method, we connect clusters depending on the data density between two cluster centroids. Moreover, our proposed visualization is directly optimized to respect the density tree, while PAGA injects graph information only at the initialization of the visualization.

**Graph and visualization:** MONOCLE 2 (Qiu *et al.*, 2017) is more similar to our method, as it provides both a visualization and a hierarchical graph structure on a vector quantization of the data. The tree in MONOCLE 2 is inferred in conjunction with the embedding, while we learn it as a first step in high-dimensional space and consider the data density explicitly. As a result, our density tree depends only on the biological data but not the embedding initialization or dimension. MONOCLE 2 is conceptually promising, but empirically found to be often inferior to other methods, confer (Moon *et al.*, 2019). Hence, we did not compare to MONOCLE 2.

**Visualization only:** Most visualization methods do not provide a graph representation of the data. PHATE (Moon *et al.*, 2019) is a recent approach which computes diffusion probabilities on the data before applying multidimensional scaling.

The general purpose dimension reduction methods t-SNE (Maaten and Hinton, 2008), UMAP (Becht *et al.*, 2019; McInnes *et al.*, 2020) and ForceAtlas2 (Jacomy *et al.*, 2014) are popular for visualizing scRNA-seq data. They aim to layout the *k*NN graph structure of the data with *t*-SNE focusing more on discrete clusters and ForceAtlas2 better representing the continuous structure (Böhm *et al.*, 2020). This often works well, but lacks the focus on hierarchies that our method provides. While the continuous focus of ForceAtlas2 seems apt to show differentiation processes in scRNA-seq datasets, we find that without a specific tree-prior the biologically interesting branching events are often poorly resolved.

Like density-tree biased autoencoder (DTAE), several recent methods for visualizing scRNA-seq data rely on neural networks. We describe them in the following. Many approaches are extensions of the AE (Rumelhart *et al.*, 1985), a network which encodes the data to a lower dimensional latent space from which it tries to decode the input. A prominent member of this family is DCA (Eraslan *et al.*, 2019) which replaces the usual reconstruction loss by a count-based ZINB loss and aims at denoising scRNA-seq data. Its extension scDeepCluster (Tian *et al.*, 2019) jointly trains a clustering model in latent space. SAUCIE (Amodio *et al.*, 2019) is another popular AE method and addresses multiple tasks including batch effect removal and clustering, for which it uses a binary hidden layer. In order to exploit more relational information, scGAE (Luo *et al.*, 2021) uses a graph AE based on the *k*NN graph and achieves good visualization results both for clustered and continuous scRNA-seq data, but without our inductive prior of a hierarchical embedding or our explicit focus on data density. Topological AEs (Moor *et al.*, 2020) are conceptually closest to our idea of retaining topological properties during dimension reduction. They compute the MST on all points, which produces less stable results than our density-based approach on cluster centroids.

Variational autoencoders (VAEs) (Kingma and Welling, 2013), a generative AE version, have also been explored. A popular VAE for scRNA-seq data is scVI (Lopez *et al.*, 2018), which explicitly models batch effects and library sizes. Instead, scVAE (Grønbech *et al.*, 2020) investigates likelihood functions suitable for scRNA-seq data and proposes a clustering model in latent space. DR-A (Lin *et al.*, 2020) apply adverserial training instead of the variational objective. Finally, scvis is a VAE tailored to visualization (Ding *et al.*, 2018) and uses a t-SNE-like regularization term in the latent space.

Ivis (Szubert *et al.*, 2019) employs a triplet loss function and a siamese neural network instead of an AE to preserve the nearest neighbor relations in the visualization.

Both scDeepCluster and scVAE shape the latent space into disconnected clusters, which is orthogonal to our goal of illustrating continuous developmental hierarchies. scVI, scGAE and scDeepCluster work with a latent space dimension larger than two and thus require an additional dimension reduction, typically with *t*-SNE, to visualize the data.

Neither of the pure visualization methods aims to bring out the hierarchical properties often present in scRNA-seq dataset. In particular, they do not use the data density to infer lineages. None of them provide a graph summary of the data. Our contribution, however, is to supply the user with a tree-shaped graph summarizing the hierarchies along dense lineages in the data as well as a 2D embedding that respects this tree shape.

## 3 Methods

### 3.1 Approximating the high-dimensional scRNA-seq data with a tree

To summarize the high-dimensional data in terms of a tree, the MST on the Euclidean distances is an obvious choice. This route is followed by Moor *et al.* (2020) who reproduce the MST obtained on their high-dimensional data in their low-dimensional embedding. However, scRNA-seq data can be noisy, and an MST built on all of our data is very sensitive to noise. Therefore, we first run *k*-means clustering on the original data, yielding more robust centroids for the MST construction and also reducing downstream complexity.

A problem with the Euclidean MST, illustrated in Figure 2, is that two centroids can be close in Euclidean space without having many data points between them. In such a case, a Euclidean MST would not capture the skeleton of our original data well. But it is crucial that the extracted tree follows the dense regions of the data if we want to visualize developmental trajectories of differentiating cells: a trajectory is plausible if we observe intermediate cell states and unlikely if there are jumps in the development. By preferring tree edges in high-density regions of the data, we ensure that the computed spanning tree is biologically plausible. Following this rationale, we build the maximum spanning tree on the complete graph over centroids whose edge weights are given by the density of the data along each edge instead of the MST on Euclidean distance. This results in a tree that (we believe) captures Waddington’s hypothesis better than merely considering cumulative differences in expression levels.

To estimate the support that a data sample provides for an edge, we follow Martinetz and Schulten (1994). Consider the complete graph G=(C,E) such that $C=\{c_{1},\ldots ,c_{k}\}$ is the set of centroids. In the spirit of Hebbian learning, that is, emphasizing connections that appear frequently, we count, for each edge, how often its incident vertices are the two closest centroids to any given datum.

As pointed out by Martinetz and Schulten (1994) this amounts to an empirical estimate of the integral of the density of observations across the second-order Voronoï region (defined as the set of points having a particular set of two centroids as its two nearest centroids) associated with this pair of cluster centers. Finally, we compute the maximum spanning tree over these Hebbian edge weights. Our strategy for building the tree is summarized in Algorithm 1.

Our data density-based tree follows the true shape of the data more closely than an MST based on the Euclidean distance weights, as illustrated in Figure 2. We claim this indicates it being a better choice for capturing developmental trajectories. Having extracted the tree shape in high dimensions, our goal is to reproduce this tree as closely as possible in our embedding.

**Algorithm 1** Density tree generation **Require:** High-dimensional data $X\in R^{n\times d}$

**Require:** Number of *k*-means centroids *k*

** procedure** GenerateTree(*X*, *k*)

**  **

C← kMeans(X,k) ▹ O(nkdt) with *t* the number of iterations

**  **

G=(C,E) the complete graph on our centroids

**  for** {*i*, *j*} a two-element subset of {1,…,k}  **do**  ▹ $O(k^{2})$

**   **

$d_{i,j}=0$

**  end for**

**  for**  i=1,…,|X|  **do**        ▹ O(nk)

**   **

$a\leftarrow \underset{j=1,\ldots ,k}{\text{arg min}}||x_{i}-c_{j}|{|}_{2}$ ****▹ Nearest centroid

**   **

$b\leftarrow \underset{\overset{j=1,\ldots ,k}{j\neq a}}{\text{arg min}}||x_{i}-c_{j}|{|}_{2}$ ****▹ Second nearest centroid

**   **

$d_{a,b}=d_{a,b}+1$ ****▹ Increase nearest centroids’ edge strength

**  end for**

**  **

T← MaxSpanningTree(G,d) ****▹ $O(k^{2}\;\text{log}\;k)$

**  return** *T*, *d***    **▹ Retains the density tree and the edge strengths

**end procedure**

### 3.2 Density-tree biased autoencoder

We use an AE to faithfully embed the high-dimensional scRNA-seq data in a low-dimensional space, and bias it such that the topology inferred in high-dimensional space is respected. An AE is an artificial neural network consisting of two concatenated subnetworks, the encoder *f*, which maps the input to lower-dimensional space, also called embedding space, and the decoder *g*, which tries to reconstruct the input from the lower-dimensional embedding. It can be seen as a non-linear generalization of principal component analysis (PCA). We visualize the low-dimensional embeddings $h_{i}=f(x_{i})$ and hence choose their dimension to be 2.

The AE is trained by minimizing the following loss terms, including new ones that bias the AE to also adhere to the tree structure.

#### 3.2.1 Reconstruction loss

The first term of the loss is the reconstruction loss, defined as

(1) $$L_{\text{rec}}=\text{MSE}(X,g(f(X)))=\frac{1}{N}\sum_{x_{i}\in X}||x_{i}-g(f(x_{i}))|{|}_{2}^{2}.$$

This term is the typical loss function for an AE and ensures that the embedding is as faithful to the original data as possible, forcing it to extract the most salient data features.

#### 3.2.2 Push-Pull loss

The main loss term that biases the DTAE toward the density tree is the push-pull loss. It trains the encoder to embed the data points such that the high-dimensional data density, and, in particular, the density tree, are reproduced in low-dimensional space.

We find a centroid in embedding space by averaging the embeddings of all points assigned to the corresponding *k*-means cluster in high-dimensional space. In this way, we can easily relate the centroids in high and low dimension, and will simply speak of centroids when the ambient space is clear from the context.

To reproduce the density structure in low-dimensional space, we want that the closest two high-dimensional centroids to a point $x_{i}\in X$ correspond to the two low-dimensional centroids that are closest to its embedding $h_{i}=f(x_{i})$. We denote the latter centroids by $c_{i,1}$ and $c_{i,2}$, and low-dimensional centroids that actually correspond to the closest high-dimensional centroids by $c\prime_{i,1}$ and $c\prime_{i,2}$. As long as $c\prime_{i,1},\;c\prime_{i,2}$ differ from $c_{i,1}$ and $c_{i,2}$, the encoder places *h_i_* next to different centroids than in high-dimensional space. To improve this, we want to move $c\prime_{i,1},\;c\prime_{i,2}$ and *h_i_* toward each other while separating $c_{i,1}$ and $c_{i,2}$ from *h_i_*. The following preliminary version of our push-pull loss implements this:

(2) $${\tilde{L}}_{\text{push}}(h_{i})=-{\left(||h_{i}-c_{i,1}|{|}_{2}+||h_{i}-c_{i,2}|{|}_{2}\right)}^{2}$$

 

(3) $${\tilde{L}}_{\text{pull}}(h_{i})={\left(||h_{i}-c{ʹ}_{i,1}|{|}_{2}+||h_{i}-c\prime_{i,2}|{|}_{2}\right)}^{2}$$

 

(4) $${\tilde{L}}_{\text{push-pull}}=\frac{1}{N}\sum_{x_{i}\in X}{\tilde{L}}_{\text{push}}(f(x_{i}))+{\tilde{L}}_{\text{pull}}(f(x_{i})).$$

The push loss decreases as *h_i_* and the currently closest centroids, $c_{i,1}$ and $c_{i,2}$, are placed further apart from each other, while the pull loss decreases when *h_i_* gets closer to the correct centroids, $c\prime_{i,1}$ and $c\prime_{i,2}$. Indeed, the push-pull loss term is minimized if and only if each embedding *h_i_* lies in the second-order Voronoï region of those low-dimensional centroids whose high-dimensional counterparts contain the data point *x_i_* in their second-order Voronoï region. In other words, the loss is zero precisely when we are reproducing the edge densities from high dimension in low dimension.

Note that we let the gradient flow through both the individual embeddings and through the centroids, which are means of embeddings themselves.

This naïve formulation of the push-pull loss has the drawback that it can become very small if all embeddings are nearly collapsed into a single point, which is undesirable for visualization. Therefore, we normalize the contribution of every embedding *h_i_* by the distance between the two correct centroids in embedding space. This prevents the collapsing of embeddings, and also ensures that each datapoint *x_i_* contributes equally, regardless of how far apart $c\prime_{i,1}$ and $c\prime_{i,2}$ are. The push-pull loss thus becomes

(5) $$L_{\text{push}}(h_{i})=-{\left(\frac{||h_{i}-c_{i,1}|{|}_{2}+||h_{i}-c_{i,2}|{|}_{2}}{||c\prime_{i,1}-c\prime_{i,2}|{|}_{2}}\right)}^{2}$$

 

(6) $$L_{\text{pull}}(h_{i})={\left(\frac{||h_{i}-c\prime_{i,1}|{|}_{2}+||h_{i}-c\prime_{i,2}|{|}_{2}}{||c\prime_{i,1}-c\prime_{i,2}|{|}_{2}}\right)}^{2}$$

 

(7) $$L_{\text{push-pull}}=\frac{1}{N}\sum_{x_{i}\in X}L_{\text{push}}(f(x_{i}))+L_{\text{pull}}(f(x_{i})).$$

So far, we only used the density information from high-dimensional space for the embedding, but not the extracted density tree itself. The push-pull loss in equation (7) is agnostic to the positions of the involved centroids within the density tree, only their Euclidean distance to the embedding *h_i_* matters. In contrast, the hierarchical structure is important for the biological interpretation of the data: it is much less important if an embedding is placed close to two centroids that are on the same branch of the density tree than it is if the embedding is placed between two different branches. In the first case, cells are just not ordered correctly within a trajectory, while in the second case we get false evidence for an altogether different pathway.

We tackle this problem by reweighing the push-pull loss with the geodesic distance along the density tree. The geodesic distance $d_{\text{geo}}(c_{a},c_{b})$ with $c_{a},c_{b}\in C$ is defined as the number of edges in the shortest path between *c_a_* and *c_b_* in the density tree. Centroids at the end of different branches in the density have a higher geodesic distance than centroids nearby on the same branch. By weighing the push-pull loss contribution of an embedded point by the geodesic distance between its two currently closest centroids, we focus the push-pull loss on embeddings which erroneously lie between different branches.

The geodesic distances can be computed quickly in $O(k^{2})$ via breadth first search, and this only has to be done once before training the AE.

The final version of our push-pull loss becomes

(8) $$\begin{array}{c} L_{\text{push-pull}}=\frac{1}{N}\sum_{x_{i}\in X}(d_{\text{geo}}(c_{i,1},c_{i,2})\\ \cdot \left(L_{\text{push}}(f(x_{i}))+L_{\text{pull}}(f(x_{i}))\right)). \end{array}$$

Note, that the normalized push-pull loss in Equation (7) and the geodesically reweighted push-pull loss in (8) both also get minimized if and only if the closest centroids in embedding space correspond to the closest centroids in high-dimensional space.

#### 3.2.3 Compactness loss

The push-pull loss replicates the empirical high-dimensional data density in embedding space by moving the embeddings into the correct second-order Voronoï region, which can be large or unbounded. For optimal visibility of the tree structure, an embedding should not only be in the correct second-order Voronoï region, but lie compactly around the line between its two centroids. To achieve this, we add the compactness loss, which is just another instance of the pull loss

(9) $$L_{\text{comp}}=\frac{1}{N}\sum_{x_{i}\in X}{\left(\frac{||h_{i}-c\prime_{i,1}|{|}_{2}+||h_{i}-c\prime_{i,2}|{|}_{2}}{||c\prime_{i,1}-c\prime_{i,2}|{|}_{2}}\right)}^{2}$$

 

(10) $$=\frac{1}{N}\sum_{x_{i}\in X}L_{\text{pull}}(f(x_{i})),$$

The compactness loss is minimized if the embedding *h_i_* is exactly between the correct centroids $c\prime_{i,1}$ and $c\prime_{i,2}$ and has elliptic contour lines with foci at the centroids.

#### 3.2.4 Cosine loss

Since the encoder is a powerful non-linear map, it can introduce artifactual curves in the low-dimensional tree branches. However, especially tight turns can impede the visual clarity of the embedding. As a remedy, we propose an optional additional loss term that tends to straighten branches.

Centroids at which the embedding should be straight are the ones within a branch, but not at a branching event of the density tree. The former can easily be identified as the centroids of degree 2.

Let *c* be a centroid in embedding space of degree 2 with its two neighboring centroids $n_{c,1}$ and $n_{c,2}$. The branch is straight at *c* if the two vectors $c-n_{c,1}$ and $n_{c,2}-c$ are parallel or, equivalently, if their cosine is maximal. Denoting by $C_{2}=\{c\in C\;|\;\text{deg}(c)=2\}$ the set of all centroids of degree 2, considered in embedding space, we define the cosine loss as

(11) $$L_{\text{cosine}}=1-\frac{1}{\left|C_{2}\right|}\sum_{c\in C_{2}}\frac{\left(c-n_{c,1})\cdot (n_{c,2}-c\right)}{||c-n_{c,1}|{|}_{2}\;||n_{c,2}-c|{|}_{2}}.$$

Essentially, it measures the cosine of the angles along the tree branch and becomes minimal if all these angles are zero and the branches straight.

A generalization of this criterion that deals with noisy edges in the density tree is discussed in Supplementary Material Section B.

#### 3.2.5 Complete loss function

Combining the four loss terms of the preceding sections, we arrive at our final loss

(12) $$L=\lambda_{\text{rec}}L_{\text{rec}}+\lambda_{\text{push-pull}}L_{\text{push-pull}}+\lambda_{\text{comp}}L_{\text{comp}}+\lambda_{\;\text{cos}\;}L_{\;\text{cos}\;}.$$

The relative importance of the loss terms, especially of $L_{\text{comp}}$ and $L_{\;\text{cos}\;}$, which control finer aspects of the visualization, might depend on the use-case. In practice, we found $\lambda_{\text{rec}}=\lambda_{\text{push-pull}}=\lambda_{\text{comp}}=1$ and $\lambda_{\;\text{cos}\;}=50$ to work well. This configuration reduces the number of weights to adjust from four to one.

An ablation study of the different losses’ contribution is available in Supplementary Material Section C. Its main conclusion is that while the push-pull loss and reconstruction loss are sufficient to obtain satisfactory results, the addition of the compactness and cosine loss helps to improve the visualizations further and facilitates reproducibility. Empirically, we found that adding the compactness loss without the cosine loss sometimes leads to discontinuous embeddings. The two loss terms should therefore be added or omitted jointly.

If the default loss weights are not satisfactory, we recommend adjusting the cosine loss weight first. To understand how changing the loss parameters may affect the results, please refer to the qualitative results in the ablation study.

### 3.3 Training procedure

Firstly, we compute the *k*-means centroids, the edge densities, the density tree, and geodesic distances. This has to be done only once as an initialization step. Secondly, we pretrain the AE with only the reconstruction loss via stochastic gradient descent on minibatches. This provides a warm start for finetuning the AE with all losses in the third step.

During finetuning, all embedding points are needed to compute the centroids in embedding space. Therefore, we perform full-batch gradient descent during finetuning. For algorithmic details regarding the training procedure, confer to Supplementary Algorithm S1.

We always used *k *=* *50 centroids for *k*-means clustering in our experiments. This number needs to be high enough so that the tree yields a skeleton of the data, but not so high that the density loses its meaning. *k *=* *50 is a default value that works well in a variety of scenarios. Our AE always has a bottleneck dimension of 2 for visualization. In the experiments, we used layers of the following dimensions d(inputdimension),2048,256,32,2,32,256,2048,d. This results in symmetrical encoders and decoders with four layers. While not necessary in our experiments, if a lighter network is desired, we recommend applying PCA first to reduce the number of input dimensions, or to filter out more genes during the preprocessing. We omitted hidden layers of dimension larger than the input. We use fully connected layers and ReLU activations after every layer but the last encoder and decoder layer and employ the Adam (Kingma and Ba, 2017) optimizer with learning rate $2\times 10^{-4}$ for pretraining and $1\times 10^{-3}$ for finetuning unless stated otherwise. We used a batch size of 256 for pretraining in all experiments. We relied on PyTorch (Paszke *et al.*, 2019) and Higra (Perret *et al.*, 2019) for our implementation.

## 4 Results

In this section, we show the performance of our method on toy and real scRNA-seq datasets and compare it to a vanilla AE, as well as to the popular non-parametric methods PCA, Force Atlas 2, UMAP and PHATE and to the most prevalent neural network-based approaches, SAUCIE, DCA and scVI. For all network-based approaches, we choose a bottleneck of dimension 2 to directly use them for visualization.

### 4.1 Phate generated data

We applied our method to an artificial dataset created with the library published alongside Moon *et al.* (2019), to demonstrate its functionality in a controlled setting. We generated a toy dataset whose skeleton is a tree with one backbone branch and 9 branches emanating from the backbone, consisting in total of 10 000 points in 100 dimensions. The generated dataset is available alongside the source code.

We pretrained for 150 epochs with a learning rate of $10^{-3}$ and finetuned for another 150 epochs with a learning rate of $10^{-2}$.

**Fig. 3.**
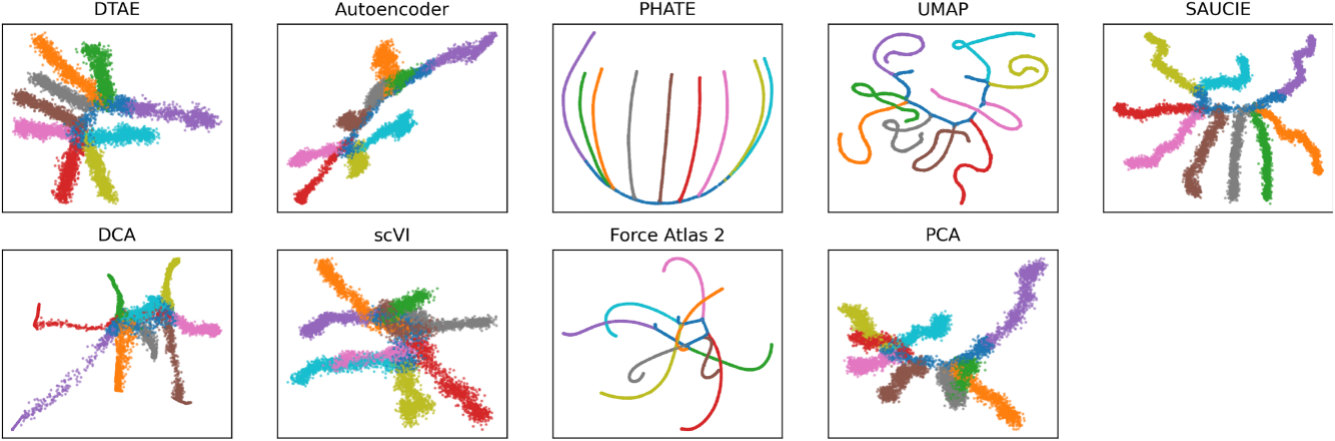
Results obtained using data generated by the PHATE library. Branches are colored by groundtruth labels

Figure 3 shows the visualization results. The finetuning significantly improves the results of the pretrained AE, whose visualization collapses the gray and green branch onto the blue branch. All methods other than DCA, scVI and PCA achieve satisfactory results that make the true tree structure of the data evident. While PHATE, UMAP and Force Atlas 2 produce overly crisp branches compared to the PCA result, the reconstruction loss of our AE guards us from collapsing the branches into lines. PHATE appears to overlap the cyan and yellow branches near the backbone, and UMAP introduces artificially curved branches. scVI collapses the green and brown as well as the pink and cyan branches together, giving hard to interpret visualizations. The results on this toy dataset demonstrate that our method can embed high-dimensional hierarchical data into 2D and emphasize its tree structure while avoiding to collapse too much information compared to state-of-the-art methods. In our method, all branches are easily visible.

### 4.2 Endocrine pancreatic cell data

We evaluated our method on the data from Bastidas-Ponce *et al.* (2019). It represents endocrine pancreatic cells at different stages of their development and consists of gene expression information for 36 351 cells and 3999 genes. Preprocessing information can be found in Bastidas-Ponce *et al.* (2019). We pretrained for 300 epochs and used 250 epochs for finetuning.

**Fig. 4.**
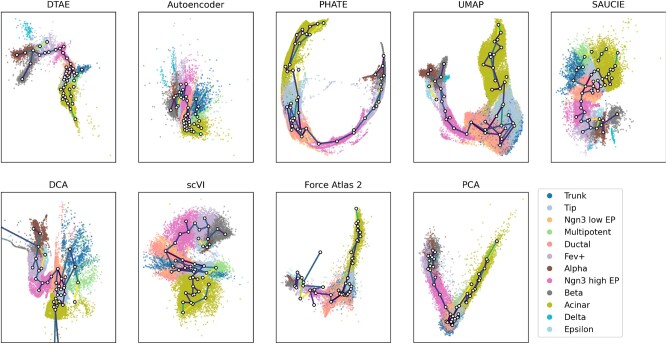
Pruned density tree superimposed over embeddings of the endocrine pancreatic cell dataset, colored by cell subtypes. We use finer labels for the endocrine cells. Darker edges represent denser edges. Only edges with more than 100 points contributing to them are plotted here

Figure 4 and Supplementary Figure S5 depict visualizations of the embryonic pancreas development with different methods. Our method can faithfully reproduce the tree structure of the data, especially for the endocrine subtypes. The visualized hierarchy is biologically plausible, with a particularly clear depiction of the *α*-, *β*- and *ε*-cell branches and a visible, albeit too strong, separation of the *δ*-cells. This is in agreement with the results from Bastidas-Ponce *et al.* (2019). UMAP also performs very well and attaches the *δ*-cells to the main trajectory. However, the *α*- and *β*-cell branches are not as prominent as in DTAE. PHATE does not manage to separate the *δ*- and *ε* cells discernibly from the other endocrine subtypes. As on toy data in Figure 3, it produces overly crisp branches for the *α*- and *β* cells. PCA mostly overlays all endocrine subtypes. All methods but the vanilla AE show a clear branch with tip and acinar cells and one via EP and Fev+ cells to the endocrine subtypes, but only DTAE, DCA, SAUCIE and scVI manage to also hint at the more generic trunk and multipotent cells from which these two major branches emanate. However, SAUCIE, DCA and scVI fail to produce a meaningful separation between the *α*- and *β*-cell branches. The ductal and Ngn3 low EP cells overlap in all methods.

It is worth noting that the AE alone was not able to visualize meaningful hierarchical properties of the data. However, the density tree-biased finetuning in DTAE made this structure evident, highlighting the benefits of our approach.

In Figure 4, we overlay DTAE’s embedding with a pruned version of the density tree and see that the visualization closely follows the tree structure around the differentiated endocrine cells. This combined representation of low-dimensional embedding and overlaid density tree further facilitates the identification of branching events, most notably for the *α*- and *β* cells, and shows the full power of our method. It also provides an explanation for the apparent separation of the *δ* cells. Since there are relatively few *δ* cells, they are not represented by a distinct *k*-means centroid.

Our method places more *k*-means centroids in the dense region in the lower right part of DTAE’s panel in Figure 4 than is appropriate to capture the trajectories, resulting in many small branches. Fortunately, this does not result in an exaggerated tree-shaped visualization that follows every spurious branch, which we hypothesize is thanks to the successful interplay between the tree bias and the reconstruction aim of the AE: If the biological signal encoded in the gene expressions can be reconstructed by the decoder from an embedding with enhanced hierarchical structure, the tree-bias shapes the visualization accordingly. Conversely, an inappropriate tree-shape is prevented if it would impair the reconstruction. Overall, the density tree recovers the pathways identified in Bastidas-Ponce *et al.* (2019) to a large extent. Only the trajectory from multipotent via tip to acinar cells includes an unexpected detour via the trunk and ductal cells, which the AE mends by placing the tip next to the multipotent cells.

The density tree also provides useful information in conjunction with other dimension reduction methods. In Figure 4, we overlay their visualizations with the pruned density tree by computing the centroids in the respective embedding spaces according to the *k*-means cluster assignments. The density tree can help to find branching events and gain insights into the hierarchical structure of the data that is visualized with an existing dimension reduction method. For instance, together with the density tree, we can identify the *ε* cells as a separate branch and find the location of the branching event into different endocrine subtypes in the UMAP embedding.

### 4.3 T-cell infection data

We further applied our method to T-cell data of a chronic and an acute infection, which was shared with us by the authors of Cerletti *et al.* (2020). The data were preprocessed using the method described in Zheng *et al.* (2017), for more details confer Cerletti *et al.* (2020). It contains gene expression information for 19 029 cells and 4999 genes. While we used the combined dataset to fit all dimension reduction methods, we only visualize the 13 707 cells of the chronic infection for which we have phenotype annotations from Cerletti *et al.* (2020) allowing us to judge visualization quality from a biological viewpoint. We pretrained for 600 epochs and used 250 epochs for finetuning.

**Fig. 5.**
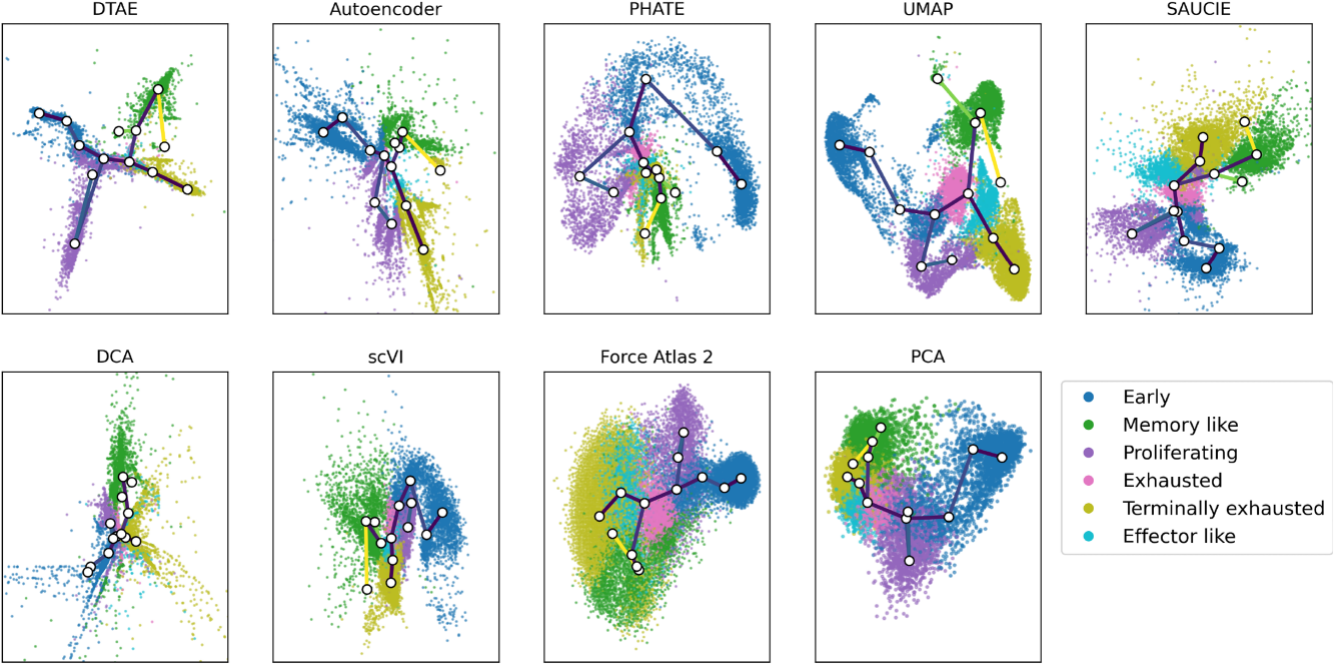
Pruned density tree superimposed over embeddings of the chronic part of the T-cell data, colored by phenotypes. Darker edges represent denser edges. Only edges with more than 100 points contributing to them are plotted here

Figure 5 and Supplementary Figure S6 demonstrate that our method makes the tree structure of the data clearly visible. The visualized hierarchy is also biologically significant: The two branches on the right correspond to the memory-like and terminally exhausted phenotypic states, which are identified as the main terminal fates of the differentiation process in Cerletti *et al.* (2020). Furthermore, the purple branch at the bottom contains the proliferating cells. Since the cell cycle affects cell transcription significantly, those cells are expected to be distinct from the rest.

It is encouraging that DTAE makes the expected biological structure apparent even without relying on known marker genes or differential cell expression, which were used to obtain the phenotypic annotations in Cerletti *et al.* (2020).

Interestingly, our method places the branching event toward the memory-like cells in the vicinity of the exhausted cells, as does UMAP, while Cerletti *et al.* (2020) recognized a trajectory directly from the early stage cells to the memory-like fate. The exact location of a branching event in a cell differentiation process is difficult to determine precisely. We conjecture that fitting the dimensionality reduction methods on the gene expression measurements of cells from an acute infection in addition to those from the chronic infection analyzed in Cerletti *et al.* (2020) provided additional evidence for the trajectory via exhausted cells to the memory-like fate. Unfortunately, an in-depth investigation of this phenomenon is beyond the scope of this methodological paper.

The competing methods expose the tree structure of the data less obviously than DTAE. The finetuning significantly improves the results from the AE, which shows no discernible hierarchical structure. PHATE separates the early cells, proliferating cells and the rest. But its layout is very tight around the biologically interesting branching event toward memory-like and terminally exhausted cells. PCA exhibits only the coarsest structure and fails to separate the later states visibly. The biological structure is decently preserved in the UMAP visualization, but the hierarchy is less apparent than in DTAE. SAUCIE, scVI and Force Atlas 2 produce results that are very similar to PCA, with later states that are hard to distinguish. DCA produces results that are very similar to the vanilla AE, where even though the later states are visible, there is a significant amount of noise in the embedding, making the analysis difficult. Overall, our method outperforms the other visualization methods on this dataset.

In Figure 5, we have overlaid our embedding with a pruned version of the density tree and see that DTAE’s visualization indeed closely follows the tree structure. It is noteworthy that even the circular behavior of proliferation cells is accurately captured by a self-overlaid branch, although our tree-based method is not directly designed to extract circular structure.

Figure 5 also shows the other dimension reduction methods in conjunction with the pruned density tree. Reassuringly, we find that all methods embed the tree in a plausible way, i.e. without many self-intersections or oscillating branches. This is evidence that our density tree indeed captures a meaningful tree structure of the data. As for the endocrine pancreas dataset, the density tree can enhance hierarchical structure in visualizations of existing dimension reduction methods. It, for example, clarifies in the UMAP plot that the pathway toward the terminally exhausted cells is via the exhausted and effector like cells and not directly via the proliferating cells.

### 4.4 Quantitative analysis

The purpose of a visualization method is to make the most salient, qualitative properties of a dataset visible. Nevertheless, a quantitative evaluation can support the comparison of visualization methods and provide evidence that the data and its visualization are structurally similar. Unfortunately, there is to our knowledge no consensus as to which metric aligns with practitioners’ notion of a useful visualization. Hence, any single metric cannot validate the quality of a method. This is why it is important to use multiple metrics, so that one can hope for a more reliable result.

We selected eight different metrics, some of which have been employed to judge visualization methods before (Becht *et al.*, 2019; Kobak and Berens, 2019; Moon *et al.*, 2019). The first group of metric considers the local structure. We compute the Adjusted Rand Index (ARI) between a *k*-means clustering in high and low dimension and the number of correct neighbors in the *k*-NN graph in high and low dimension. The next category is global metrics, which rely on distance preservation. Euclidean distances are computed in low dimension and Euclidean or geodesic distances are computed in high dimension. Then correlations are computed between those distances. Finally, we use Voronoï diagram-based metrics. First or second order Voronoï diagrams on the *k*-means centroids are computed using the *k*-means assignments to obtain the seeds in low-dimensional space. Then the ratio of points placed in the correct Voronoï region is computed. When using the second order Voronoï diagram with *k *=* *50, there is a bias toward DTAE since we optimize this criterion. For local and Voronoï diagram-based metrics, we have to adjust a parameter *k* (either for *k*-means clustering or for a *k*-NN graph). We vary the value of *k* between 10 and 100 with a step of 10 and report the area under the curve.

We report results aggregated on all three datasets in Table 1 and full results are available in Supplementary Table S5. This aggregation makes it easier to deduce general patterns of performance among multiple datasets. From the results on all datasets, we can clearly see that DTAE outperforms other methods on Voronoï diagram-based metrics, in part due to the bias toward them for *k *=* *50. On local metrics, DTAE achieves the best performance on ARI, followed closely by SAUCIE. However, for *k*-NN preservation UMAP performs better than other methods by a significant margin which is consistent with the criterion it optimizes (Damrich and Hamprecht, 2021). For Euclidean distance preservation, AE-based methods perform the best, with no clear winner overall. For geodesic distance preservation, PCA performs the best, even though it produced poor visualizations. This is in line with previous findings (Kobak and Berens, 2019). Most other methods obtained very similar performance on this metric, making it hard to conclude that any method performs better than another.

**Table 1.** Relative quantitative performances averaged over all studied datasets

Type of metric	Local		Global				Voronoi		
Metric	ARI	k-NN	Euclidean		Geodesic		1st order	2nd order	All
			Pearson	Spearman	Pearson	Spearman			
DTAE (Ours)	**93.75**	48.70	85.51	72.91	82.39	87.19	**98.24**	**94.21**	**82.86**
AE	74.83	70.96	87.41	77.20	70.16	73.23	89.83	58.43	75.26
PHATE	84.76	73.48	45.43	46.04	74.15	78.45	85.27	44.04	66.45
UMAP	78.88	**87.75**	53.42	54.31	79.40	80.12	83.31	55.94	71.64
SAUCIE	89.99	67.43	82.22	78.50	84.03	85.41	96.43	78.58	82.83
DCA	49.79	64.37	76.54	**90.95**	40.40	65.92	63.26	49.33	62.57
scVI	74.80	54.30	**87.82**	67.68	75.45	82.75	86.42	57.77	73.37
Force Atlas 2	72.88	72.23	37.28	48.06	35.67	76.65	77.27	43.27	57.91
PCA	60.40	40.78	73.42	66.02	**96.44**	**96.40**	80.76	56.82	71.38

*Note*: For each metric, we give the best performing method a value of 100 and scale other results proportionally. The metrics are described in Section 4.4 and higher values indicate better performance. The rightmost column contains the average relative performance over all metrics. DTAE and SAUCIE have the best performance overall, with DTAE excelling in Voronoï metrics and ARI. Bold values indicate the best performance.

In order to more easily compare methods, aggregated performances over all metrics are reported in the rightmost column of Table 1. This aggregation makes it easier to evaluate the overall performance of a method when using a wide variety of criteria. We chose the arithmetic mean to combine the results for simplicity’s sake. From this, we can see that DTAE and SAUCIE perform significantly better than other methods, with DTAE surpassing SAUCIE by a small margin. However, from a qualitative point of view, DTAE produced superior visualizations compared to SAUCIE, as discussed previously.

Overall, DTAE produced excellent results both from a quantitative and qualitative point of view, highlighting its usefulness as a visualization method for tree-shaped data.

## 5 Limitations

### 5.1 Hierarchy assumption

**Fig. 6.**
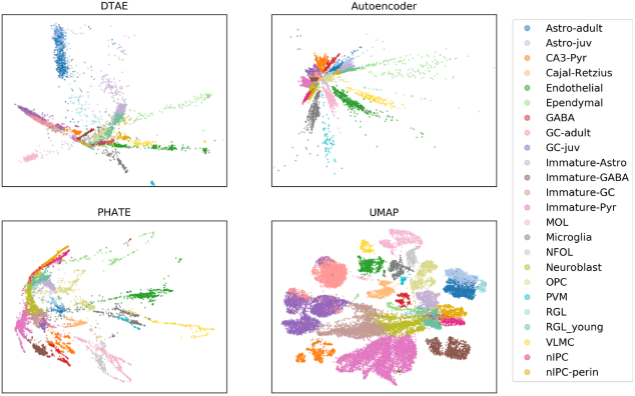
Failure case: Highly clustered data violates our underlying assumption of a tree structure. Dentate gyrus data from Hochgerner *et al.* (2018) with clusters colored by groundtruth cluster assignments

Our method is tailored to Waddington’s hierarchical structure assumption of developmental cell populations, in which the highest data density is along the developmental trajectory. It produces convincing results in this setting as shown above. However, if the assumption is violated, for instance because the dataset contains multiple separate developmental hierarchies or a mixture of hierarchies and distinct clusters of fully differentiated cell fates, the density tree cannot possibly be a faithful representation of the dataset. Indeed, in such a case, our method yields a poor result. As an example, confer Figure 6 with visualizations of the dentate gyrus dataset from Hochgerner *et al.* (2018), preprocessed according to Zheng *et al.* (2017). This dataset consists of a mostly linear cell trajectory and several distinct clusters of differentiated cells, and consequently does not meet our model’s assumption. Indeed, DTAE manages to only extract some linear structures, but overall fails on this dataset, similarly to PHATE. UMAP seems to produce the most useful visualization here.

One could adapt our method by extracting a forest of disconnected density trees by cutting edges below a density threshold. However, if little is known *a priori* about the structure of the dataset, a more general dimension reduction method might be preferable for initial data exploration.

### 5.2 Neural network limitations

Artificial neural networks are powerful non-linear functions that can produce impressive results. Unfortunately, they require the choice of a number of hyperparameters, such as the dimension of the hidden layers and the learning rate, making them less end-user friendly than their classical counterparts.

## 6 Conclusion

We have introduced a new way of capturing the hierarchical properties of scRNA-seq data of a developing cell population with a density-based MST. This tree is a hierarchical representation of the data that places edges in high-density regions and thus captures biologically plausible trajectories. The density tree can be used to inform any dimension reduction method about the hierarchical nature of the data.

Moreover, we used the density tree to bias an AE and were thus able to produce promising visualizations exhibiting clearly visible tree structure both on synthetic and real-world scRNA-seq data of developing cell populations.

## Funding

This work was supported, in part, by the Informatics for Life funded by the Klaus Tschira Foundation.

*Conflict of Interest*: none declared.

## Supplementary Material

btac249_Supplementary_Data
