# Supplementary material for: Visualizing hierarchies in scRNA-seq data using a density tree-biased autoencoder
Source: Bioinformatics. 2022 Jun 27;38(Suppl 1):i316–24. doi: 10.1093/bioinformatics/btac249 (PMC9235514; doi:10.1093/bioinformatics/btac249)
Supplement: btac249_Supplementary_Data [file btac249_supplementary_data.pdf]

Supplementary material for:
Visualizing hierarchies in scRNA-seq data using a density tree-biased autoencoder

Quentin Garrido [1,5] Sebastian Damrich [1] Alexander Jäger [1] Dario Cerletti [2,3]
Manfred Claassen [4] Laurent Najman [5] Fred A. Hamprecht [1]

[1] HCI/IWR, Heidelberg University, Germany
[2] Institute of Molecular Systems Biology, ETH Zürich, Switzerland
[3] Institute of Microbiology, ETH Zürich, Switzerland
[4] Internal Medicine I, University Hospital Tübingen, University of Tübingen, Germany
[5] Université Gustave Eiffel, CNRS, LIGM, F-77454 Marne-la-Vallée, France

A. Training loop algorithm

Algorithm S1 Training loop

Require: Autoencoder $(g \circ f)_\theta$
Require: Pretraining epochs n_p, batch size b and learning rate α_p
Require: Finetuning epochs n_f and learning rate α_f
Require: Weight parameters for the loss $\lambda_{\text{rec}}, \lambda_{\text{push-pull}}, \lambda_{\text{comp}}, \lambda_{\text{cos}}$

$T, C, C_2, d_{geo} \leftarrow$ INITIALIZATION(X)
$\#Pretraining$
for $t = 0, 1, \ldots, n_p$ **do**
    for $i = 0, 1, \ldots, n_p/b$ **do**
        Sample a minibatch m from X
        $\hat{m} \leftarrow g(f(m))$
        $\mathcal{L} \leftarrow \mathcal{L}_{\text{rec}}$
        $\theta^{t+1} \leftarrow \theta^t - \alpha_p \nabla \mathcal{L}$
    end for
end for
$\#Finetuning$
for $t = n_p, \ldots, n_p + n_f$ **do**
    $h \leftarrow f(X)$
    $\hat{X} \leftarrow g(h)$
    $\mathcal{L} \leftarrow \lambda_{\text{rec}}\mathcal{L}_{\text{rec}} + \lambda_{\text{push-pull}}\mathcal{L}_{\text{push-pull}} + \lambda_{\text{comp}}\mathcal{L}_{\text{comp}} + \lambda_{\text{cos}}\mathcal{L}_{\text{cos}}$
    $\theta^{t+1} \leftarrow \theta^t - \alpha_f \nabla \mathcal{L}$
end for

B. Cosine loss generalization

The definition of a vertex' degree in a graph as the number of incident edges to it is not perfect, as it does not take into account the noisiness of the graph. On real datasets, we may have stray clusters which lead to noisy edges in the density graph. These usually manifest as edges with only one point contributing to them in high dimension. This leads to vertices with an effective degree of 2 that have a higher degree due to these noisy edges, and are thus ignored by the cosine loss.

To remedy this, we introduce a different definition of degree. We consider a threshold $t \in [0, 100]$ and define the degree of a vertex as the smallest number of incident edges that account for $t\%$ of all points contributing to the vertex's incident edges. As t gets closer to a hundred, we converge to the original definition of degree.

More formally put, consider a weighted graph $G = (V, E, W)$ and a function Γ that returns incident edges to a given vertex sorted by their weights. This alternative definition of a vertex's degree is then:

$$\deg(v, t) = \min_{n=1\ldots|\Gamma(v)|} n \quad \text{s.t.} \quad \frac{\sum_{i=1}^{n} W_{\Gamma(v)_i}}{\sum_{j=1}^{|\Gamma(v)|} W_{\Gamma(v)_j}} \geq \frac{t}{100}$$

We can clearly see that when $t = 100$ we obtain the classical definition of degree. As this generalization has not improved the visualization quality drastically, we opted for the simpler version of the cosine loss in the main paper.

C. Ablation study

In order to better visualize the contributions of each element of our method, we conducted an ablation study of the different loss parameters and evaluated their impact both qualitatively and quantitatively.

C.1. Loss parameters

The first phenomenon that is studied is the influence of dropping loss terms entirely. The reconstruction loss is always kept since it is necessary for the embeddings to contain salient information about the data. Not all combinations of loss parameters will be studied, but only those that should be interesting (for example, using only the cosine loss does not make much sense, so it is not an interesting scenario).

We will not study the influence of the weights for every loss since the default weights of 1 lead to good performance and this configuration significantly reduces the dimension of the hyperparameter space. All experiments are described in table S1.

The performance will be evaluated both qualitatively and quantitatively on all three discussed datasets to demonstrate as clearly as possible the impact of every loss term.

Experiment	$\mathcal{L}_{\text{rec}}$	$\mathcal{L}_{\text{push-pull}}$	$\mathcal{L}_{\text{comp}}$	$\mathcal{L}_{\text{cos}}$ (weight)
A	✓			
B	✓		✓	
C	✓	✓		
D	✓	✓	✓	
E	✓	✓		✓(50)
F	✓	✓	✓	✓(50)

Table S1: List of loss parameters for our ablations.

As can be seen in figures S1,S2 and S3, the compactness loss alone is not sufficient to obtain a good representation since it has no repulsive force. The reconstruction loss helps to avoid a total collapse but is not sufficient to prevent a partial collapse, as visible in the endocrine pancreas and the T-cell datasets. While the push-pull loss already gives good results when used alone, since the tree structure is visible, adding the compactness loss yields embeddings in which

Type of metric	Local		Global				Voronoi	
Metric	ARI	k-NN	Euclidean		Geodesic		1st order	2nd order
			Pearson	Spearman	Pearson	Spearman		
$\lambda_{pp}=0, \lambda_{comp}=0, \lambda_{cos}=0$	34.62	19.09	0.66	0.66	0.56	0.56	70.01	38.98
$\lambda_{pp}=0, \lambda_{comp}=1, \lambda_{cos}=0$	38.54	23.20	0.80	0.79	0.74	0.73	70.44	35.24
$\lambda_{pp}=1, \lambda_{comp}=0, \lambda_{cos}=0$	48.19	25.05	0.78	0.76	0.72	0.70	**79.68**	**56.02**
$\lambda_{pp}=1, \lambda_{comp}=1, \lambda_{cos}=0$	**48.70**	**26.40**	**0.81**	0.78	0.74	0.72	79.26	55.93
$\lambda_{pp}=1, \lambda_{comp}=0, \lambda_{cos}=50$	45.54	22.71	0.80	0.77	0.74	0.72	78.94	52.97
$\lambda_{pp}=1, \lambda_{comp}=1, \lambda_{cos}=50$	46.00	24.60	**0.81**	**0.80**	**0.75**	**0.74**	78.85	53.77

(a) PHATE generated dataset.

Metric	ARI	k-NN	Euclidean		Geodesic		1st order	2nd order
			Pearson	Spearman	Pearson	Spearman		
$\lambda_{pp}=0, \lambda_{comp}=0, \lambda_{cos}=0$	34.52	**4.17**	**0.81**	**0.85**	0.57	0.62	65.37	30.83
$\lambda_{pp}=0, \lambda_{comp}=1, \lambda_{cos}=0$	24.88	2.32	0.65	0.69	0.53	0.59	46.30	11.11
$\lambda_{pp}=1, \lambda_{comp}=0, \lambda_{cos}=0$	43.07	3.07	0.77	0.79	**0.71**	0.77	**73.79**	**50.68**
$\lambda_{pp}=1, \lambda_{comp}=1, \lambda_{cos}=0$	**44.69**	2.93	0.73	0.79	0.66	0.75	72.95	46.75
$\lambda_{pp}=1, \lambda_{comp}=0, \lambda_{cos}=50$	35.64	2.77	0.73	0.75	0.70	0.75	68.44	38.82
$\lambda_{pp}=1, \lambda_{comp}=1, \lambda_{cos}=50$	39.79	2.85	0.71	0.74	**0.71**	**0.78**	69.24	38.04

(b) Endocrine pancreas dataset.

Metric	ARI	k-NN	Euclidean		Geodesic		1st order	2nd order
			Pearson	Spearman	Pearson	Spearman		
$\lambda_{pp}=0, \lambda_{comp}=0, \lambda_{cos}=0$	29.24	**2.20**	0.40	**0.33**	0.40	0.42	35.17	4.50
$\lambda_{pp}=0, \lambda_{comp}=1, \lambda_{cos}=0$	40.65	1.24	0.15	0.17	0.20	0.20	28.75	2.75
$\lambda_{pp}=1, \lambda_{comp}=0, \lambda_{cos}=0$	29.72	1.28	**0.42**	0.26	0.36	0.39	**47.19**	**18.63**
$\lambda_{pp}=1, \lambda_{comp}=1, \lambda_{cos}=0$	**45.55**	1.15	0.38	0.23	0.35	0.38	37.71	16.29
$\lambda_{pp}=1, \lambda_{comp}=0, \lambda_{cos}=50$	29.24	1.23	0.37	0.24	**0.42**	**0.44**	44.73	12.16
$\lambda_{pp}=1, \lambda_{comp}=1, \lambda_{cos}=50$	37.25	1.15	0.31	0.19	0.40	0.41	38.41	12.85

(c) T-cells dataset.

Table S2: Quantitative results in different scenarios for DTAE's loss weights.

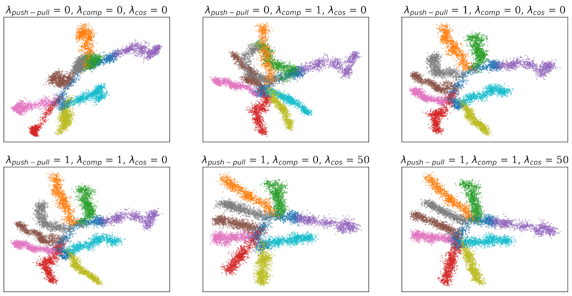

Figure S1: Results of the ablations on the PHATE generated dataset, colored by groundtruth clusters.

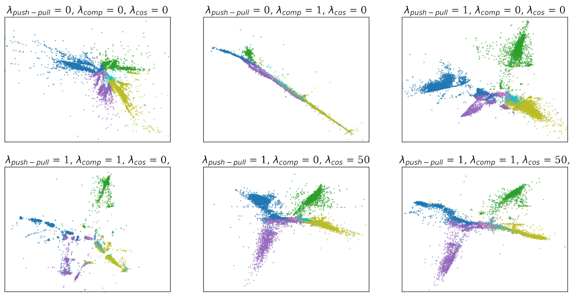

Figure S2: Results of the ablations on the T-cell dataset, colored by phenotypes.

the points lie compactly along the tree. Without the cosine loss, however, this combination can lead to sparse representation due to the fact that seeds of second order Voronoï cells do not necessarily lie in their cell. This means that points will not necessarily be spread out along the line between two centroids but only lie inside the intersection of

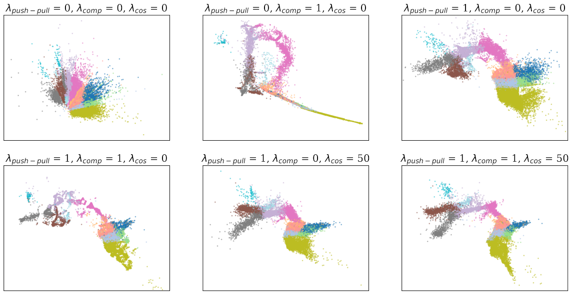

Figure S3: Results of the ablations on the endocrine pancreas dataset, colored by cell types.

	Rel. Perf.
$\lambda_{pp} = 0, \lambda_{comp} = 0, \lambda_{cos} = 0$	81.27
$\lambda_{pp} = 0, \lambda_{comp} = 1, \lambda_{cos} = 0$	67.60
$\lambda_{pp} = 1, \lambda_{comp} = 0, \lambda_{cos} = 0$	**92.04**
$\lambda_{pp} = 1, \lambda_{comp} = 1, \lambda_{cos} = 0$	90.66
$\lambda_{pp} = 1, \lambda_{comp} = 0, \lambda_{cos} = 50$	87.24
$\lambda_{pp} = 1, \lambda_{comp} = 1, \lambda_{cos} = 50$	86.99

Table S3: Relative performance out of a hundred over all datasets and metrics.

the line between the two centroids and their second order Voronoï cell, which may be much smaller than the full line between the centroids. Using only the push-pull and cosine loss can lead to satisfying results, but the embedding is more spread out than with the compactness loss. Adding the cosine loss makes all the results cleaner and helps with the density of the point cloud. This effect is discussed in the next section.

From a quantitative point of view, adding all of these losses leads to worse performances than just using the push-pull loss alone. Since the compactness and cosine losses are designed with visualization in mind, they can alter the fidelity of the embedding. For example, making the points tighter along the density tree will lead to pairwise distances that are preserved more poorly, which is an effect that we indeed observe in the global metrics in table S2.
Nonetheless, when looking at aggregated performances in table S3 we can see that all experiments except when using the compactness loss alone still perform comparatively. As such, the increase in qualitative performance stemming from the addition of losses is not done at the expense of the preservation of the data's intrinsic structure. In particular, the push-pull loss alone drastically improves the visualization not only qualitatively, but also quantitatively.

C.2. Cosine loss weight

A parameter that is interesting to study in more detail is the cosine loss weight. While a lot of the other losses have a significant impact on the embeddings, the cosine loss is mostly cosmetic, and it is important to understand its behavior for low and high weights. The cosine loss weight will only be studied on the T-cell dataset, since it is enough to demonstrate its impact on quantitative and qualitative results.

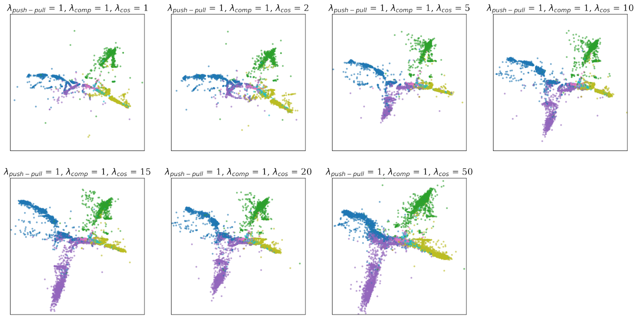

Figure S4: Results obtained on the chronic infection subset of the T-cell dataset when varying the cosine loss weight, colored by phenotypes.

As can be seen in figure S4 the cosine loss straightens the branches for every weight as intended. However, with higher weights, it also has a density regularizing effect. As its weight increases, we obtain a more homogeneous and less clumped point cloud. While there is no clear explanation for this behavior, a hypothesis is that the higher weight means that this criterion will be optimized with higher priority during the finetuning. Since the pretraining produces dense embedding and this cosine loss has no incentive to produce sparse embeddings, this denser structure is kept during training. On the contrary, the push-pull loss can have a sparsifying effect, since the seeds of second order Voronoï cells do not necessarily lie in their cells. When the cosine loss weight is smaller, this loss is optimized with higher priority, which would lead to the sparser embeddings. All of this is intimately linked to the dynamics of neural network training and not only to minimizers of each criterion, making a precise study of this process highly complex.

From a quantitative point of view, a slight decrease in performance is visible in table S4 for all metrics except for the preservation of geodesic distances or of first order Voronoï diagrams. As a result, the overall performance decreases noticeably when increasing the cosine loss weight, see the rightmost column in table S4.

This again illustrates the trade-offs between quantitative and qualitative performance, where even though a method performs slightly worse quantitatively, it might still produce results that are easier to interpret for humans.

Type of metric	Local		Global				Voronoi		All
			Euclidean		Geodesic				
Metric	ARI	k-NN	Pearson	Spearman	Pearson	Spearman	1st order	2nd order	
$\lambda_{cos} = 1$	**45.83**	1.15	**0.37**	**0.24**	0.38	0.39	37.30	16.36	**95.68**
$\lambda_{cos} = 2$	44.77	1.11	0.35	**0.24**	0.40	0.40	37.90	**16.39**	95.34
$\lambda_{cos} = 5$	45.22	1.09	**0.37**	0.20	0.40	0.45	37.38	14.12	93.30
$\lambda_{cos} = 10$	44.29	1.12	0.35	0.18	0.42	**0.46**	36.40	13.66	91.83
$\lambda_{cos} = 15$	43.50	1.14	0.29	0.15	**0.44**	**0.46**	**38.78**	14.59	90.28
$\lambda_{cos} = 20$	43.08	**1.17**	0.33	0.17	0.42	**0.46**	37.43	14.41	91.74
$\lambda_{cos} = 50$	37.25	1.15	0.31	0.19	0.40	0.41	38.41	12.85	87.50

Table S4: Quantitative results on the T-cells dataset when varying the cosine loss weight. The weights for the push-pull and compactness losses are set to one. The rightmost column contains the average performance over all metrics for a given method.

D. High resolution results

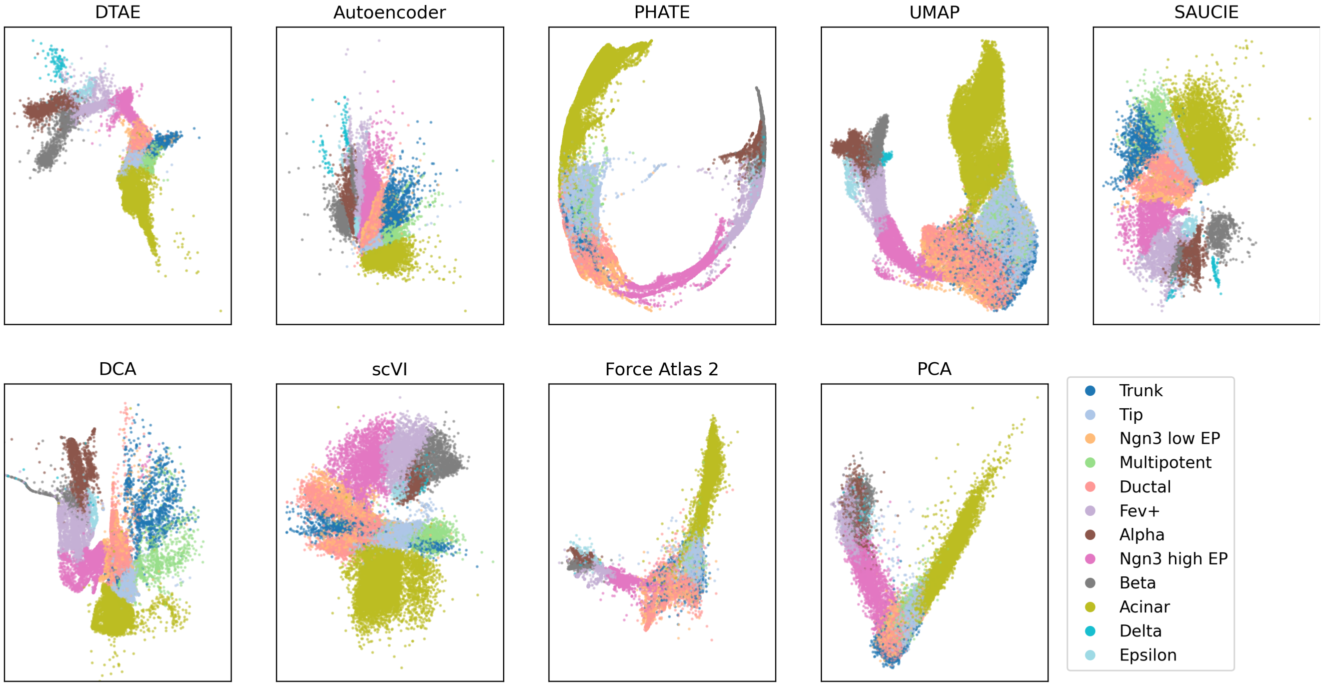

Figure S5: Results obtained on the endocrine pancreatic cell dataset, colored by cell types.

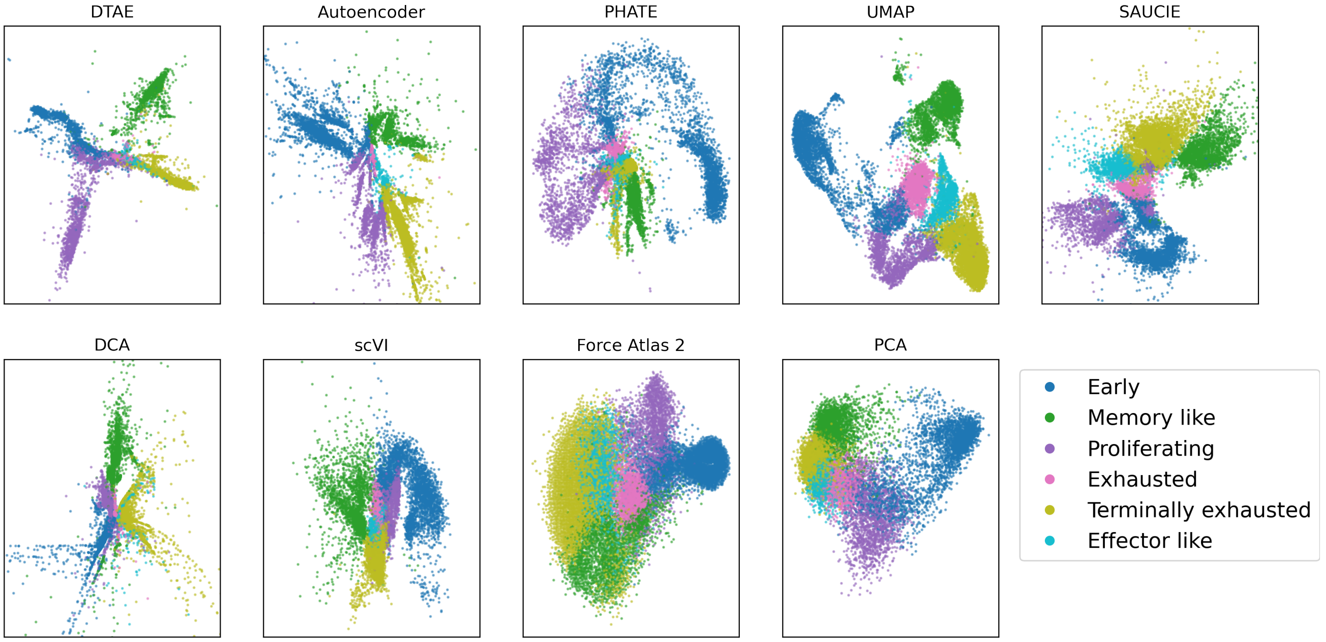

Figure S6: Results obtained on the chronic infection subset of the T-cell dataset, colored by phenotypes.

E. Complete quantitative results

Type of metric	Local		Global				Voronoi	
Metric	ARI	k-NN	Euclidean		Geodesic		1st order	2nd order
			Pearson	Spearman	Pearson	Spearman		
DTAE (Ours)	46.00	24.60	**0.81**	**0.80**	**0.75**	**0.74**	78.85	53.77
AE	34.67	19.18	0.63	0.64	0.55	0.54	70.56	38.64
PHATE	51.33	60.44	0.50	0.46	0.54	0.52	71.12	30.40
UMAP	55.13	**67.92**	0.53	0.48	0.54	0.51	75.18	46.19
SAUCIE	**56.62**	37.61	**0.81**	0.79	**0.75**	0.73	**82.98**	**65.07**
DCA	40.41	21.29	0.64	0.64	0.59	0.59	73.83	43.17
scVI	36.51	19.68	0.69	0.68	0.67	0.67	69.94	36.98
Force Atlas 2	51.85	64.38	0.59	0.55	0.56	0.54	75.79	46.44
PCA	39.87	22.36	0.77	0.74	0.67	0.66	73.14	42.53

(a) PHATE generated dataset.

Metric	ARI	k-NN	Euclidean		Geodesic		1st order	2nd order
			Pearson	Spearman	Pearson	Spearman		
DTAE (Ours)	**39.79**	2.85	0.71	0.74	0.71	0.78	69.24	**38.04**
AE	34.12	4.24	**0.81**	**0.84**	0.58	0.62	65.12	30.53
PHATE	30.92	3.70	0.64	0.65	0.71	0.78	57.22	22.27
UMAP	30.41	4.67	0.57	0.58	0.79	0.82	57.79	21.21
SAUCIE	38.94	3.69	**0.81**	0.81	0.71	0.73	**69.46**	37.93
DCA	30.93	**5.01**	0.41	0.78	0.37	0.63	64.29	27.98
scVI	32.99	3.80	0.67	0.68	0.65	0.67	61.29	27.52
Force Atlas 2	25.11	3.96	0.28	0.62	0.21	0.74	46.24	13.08
PCA	21.84	1.94	0.69	0.67	**0.87**	**0.87**	48.65	15.50

(b) Endocrine pancreas dataset.

Metric	ARI	k-NN	Euclidean		Geodesic		1st order	2nd order
			Pearson	Spearman	Pearson	Spearman		
DTAE (Ours)	**37.25**	1.15	0.31	0.19	0.40	0.41	**38.41**	**12.85**
AE	28.87	**2.17**	0.38	0.32	0.43	0.43	34.84	4.58
PHATE	32.00	1.25	-0.02	0.02	0.42	0.43	33.70	3.45
UMAP	23.41	1.52	0.11	0.21	0.46	0.44	29.24	5.28
SAUCIE	26.86	1.59	0.21	0.25	0.43	0.42	34.30	4.63
DCA	0.10	1.34	**0.45**	**0.62**	0.00	0.26	3.17	1.04
scVI	28.69	1.26	0.43	0.23	0.38	0.46	33.31	5.67
Force Atlas 2	23.83	0.93	0.02	0.01	0.05	0.41	28.39	3.09
PCA	20.82	1.10	0.18	0.16	**0.61**	**0.57**	32.30	8.27

(c) T-cells dataset.

Table S5: Full Quantitative results on all studied datasets. Metrics are described in section **??** and higher values indicate better performance.
